# Efficacy and safety of PD-1/PD-L1 inhibitor-based immune combination therapy versus sorafenib in the treatment of advanced hepatocellular carcinoma: a meta-analysis

**DOI:** 10.3389/fmed.2024.1401139

**Published:** 2024-05-02

**Authors:** Mingjin She, Yayun Wu, Mengmeng Cheng, Sanli Feng, Guizhi Li, Hui Rong

**Affiliations:** Department of Oncology, The Anhui Provincial Corps Hospital of Chinese People’s Armed Police Forces, Hefei, China

**Keywords:** PD-1, PD-L1, hepatocellular carcinoma (HCC), adverse events (AEs), immune checkpoint inhibitors, sorafenib, meta-analysis

## Abstract

**Objective:**

To systematically evaluate the safety and efficacy of PD-1/PD-L1 inhibitor-based immunotherapy (hereafter referred to as “combination immunotherapy”) compared with that of sorafenib in the treatment of hepatocellular carcinoma (HCC).

**Methods:**

Databases such as PubMed, Embase, and the Cochrane Library were searched from the date of their establishment to September 2023 to identify randomized controlled trials (RCTs) of combination immunotherapy versus sorafenib for the treatment of advanced HCC. Two reviewers independently evaluated the quality of the included studies, extracted the data, and cross-checked the information. The meta-analysis was performed using RevMan 5.3 software.

**Results:**

A total of 5 RCTs were included. The results of the meta-analysis showed the following: (1) Effectiveness. Compared to sorafenib, combination immunotherapy significantly improved overall survival (OS, HR = 0.69, 95% CI: 0.58 ~ 0.82, *p* < 0.01) and progression-free survival (PFS, HR = 0.62, 95% CI: 0.50 ~ 0.78, *p* < 0.001) in patients with advanced HCC. (2) Safety. Both groups had comparatively high incidences of adverse events (AEs), but the difference in any treatment-related adverse events was not significant between the two arms (OR = 0.98, 95% CI: 0.95 ~ 1.02, *p* = 0.34). The difference in the incidence of grade 1–2 adverse reactions was statistically significant (OR = 0.66, 95% CI = 0.49–0.90, *p* = 0.001). There were no differences in grade 3/4 TRAEs or grade 5 TRAEs (OR = 1.46, 95% CI = 0.78 ~ 2.71, *p* = 0.24; OR = 1.08, 95% CI = 0.73 ~ 1.58, *p* = 0.71).

**Conclusion:**

Combined immunotherapy can significantly prolong the OS and PFS of patients with advanced HCC without increasing the incidence of adverse effects in terms of safety, but the incidence of AEs in different systems is different.

## 1 Introduction

Hepatocellular carcinoma (HCC) accounts for more than 80% of all primary liver cancer cases (1). In recent years, immunotherapy for liver cancer has achieved remarkable curative effects (2). The US Food and Drug Administration has approved the use of two anti-PD-1 antibodies, pembrolizumab and nivolumab, as monotherapy options for the treatment of HCC (3, 4). Over the past five years, immune checkpoint inhibitors (ICIs) targeting the PD-1/PD-L1 pathway have emerged as novel and promising therapeutic agents for patients with advanced HCC. Despite this progress, monotherapy with ICIs has shown limited efficacy, with only a small proportion of HCC patients experiencing a response (5, 6). This reality has prompted clinicians to shift their focus toward combination therapies as a potential solution for HCC management.

The landscape of HCC treatment has been dramatically altered by the innovative application of ICI-based combination therapies, leading to substantial improvements in clinical outcomes for patients and marking the dawn of a new era in the management of HCC (7). The IMbrave 150 study, a phase III international multicenter clinical trial, showed the efficacy of the atezolizumab-bevacizumab combination, reporting a median overall survival (mOS) of 19.2 months, a median progression-free survival (mPFS) of 6.9 months, and an objective response rate (ORR) of 30%. Notably, the Chinese subgroup has a greater therapeutic advantage, with the combined treatment group achieving an overall survival (OS) of 24.0 months (8). The combination of atezolizumab and bevacizumab is currently considered a new standard for first-line treatment of HCC and has been approved in multiple countries worldwide. The search for effective immune-based combinations continues, as evidenced by the COSMIC-312 trial, which revealed no significant difference in OS when atezolizumab plus cabozantinib was compared with sorafenib as a first-line treatment for advanced HCC (9). Conversely, the ORIENT-32 phase II/III trial reported significant differences in both PFS and OS for patients receiving sintilimab in combination with IBI305 (a bevacizumab analog) compared to patients receiving sorafenib monotherapy (10). The HIMALAYA trial, a global multicenter, open-label phase III study, explored the use of tremelimumab in conjunction with durvalumab in patients with unresectable HCC and no prior systemic therapy, achieving an objective response rate (ORR) of 20.1% and an OS of 16.4 months, which was favorable when compared to the 13.8-month median OS observed in patients treated with sorafenib alone (11). The 2022 Chinese Society of Clinical Oncology (CSCO) Liver Cancer Guidelines now include tremelimumab in combination with durvalumab (STRIDE regimen) as a Class I expert recommendation. A phase III CARES-310 clinical study demonstrated that first-line treatment with camrelizumab combined with apatinib significantly improved both the mOS and mPFS compared with sorafenib, with respective values of 22.1 and 5.6 months, respectively, and an ORR of 25.4%, outperforming that of the sorafenib control group (12). In light of these findings, we conducted a meta-analysis to provide a comprehensive evaluation of the efficacy and safety of immune-based combination therapy compared to sorafenib monotherapy for the treatment of HCC. We aimed to systematically assess and perform a meta-analysis of existing clinical studies on immune-based combination therapy and to provide a scientific foundation for clinical decision-making.

## 2 Materials and methods

### 2.1 Search strategy

We searched the PubMed, Embase, Cochrane Library, and Web of Science databases for randomized controlled trials (RCTs) comparing immunotherapy with sorafenib for the treatment of advanced/metastatic HCC.

The search period ranged from database establishment to September 16, 2023. Only articles published in full text and written in English in peer-reviewed journals were included. This study used a combination of these words and free words for retrieval. The English search term: hepatocellular carcinoma (Mesh), HCC, Liver Neoplasms, Neoplasms, Hepatic, Neoplasms, Liver, Liver Neoplasm, Neoplasm, Liver, Hepatic Neoplasms, Hepatic Neoplasm, Neoplasm, Hepatic, Cancer of Liver, Hepatocellular Cancer, Cancers, Hepatocellular, Hepatocellular Cancers, Hepatic Cancer, Cancer, Hepatic, Cancers, Hepatic, Hepatic Cancers, Liver Cancer, Cancer, Liver, Cancers, Liver, Liver Cancers, Cancer of the Liver, Cancer, Hepatocellular, HCC; immune checkpoint inhibitors, PD-1, PD-L1, CTLA-4, Toripalimab, Sintilimab, Tislelizumab, Camrelizumab, penpulimab, serplulimab, zimberelimab, Pucotenlimab, nivolumab (Opdivo), Pembrolizumab (Keytruda), Durvalumab (Imfinzi), atezolizumab (Tecentriq), envafolimab, sugemalimab, ipilimumab (YERVOY), tremelimumab (Ticilimumab), avelumab, sorafenib, “first-line treatment”.

### 2.2 Selection criteria

#### 2.2.1 Inclusion criteria

(1) prospective phase II and III randomized controlled trials (RCTs) involving patients with advanced or metastatic HCC; (2) interventions involving comparisons of immune combination therapy with sorafenib for the treatment of HCC; and (3) outcome measures: OS, progression-free survival (PFS), complete response (CR) rate, partial response (PR) rate, and adverse events (AEs).

#### 2.2.2 Exclusion criteria

(1) lacked a control group; (2) replicated studies; (3) non-RCTs; and (4) reported outcome measures that were not relevant to the study objectives.

### 2.3 Data extraction

The extracted data included (1) basic study information (authors, year, experimental phase, country of conduct, etc.); (2) intervention details and the dosage of immunosuppressants used; (3) patient population size; and (4) outcome measures: OS, PFS, AEs, hazard ratios (HRs), and 95% confidence intervals (CIs).

The literature search and identification were independently conducted by each of the three authors. The Response Evaluation Criteria In Solid Tumors (RECIST) 1.1 criteria were utilized for assessment. The meta-analyses were conducted according to the Preferred Reporting Items for Systematic Reviews and Meta-Analyses (PRISMA) guidelines (13).

### 2.4 Risk of Bias

**Figure 1:**
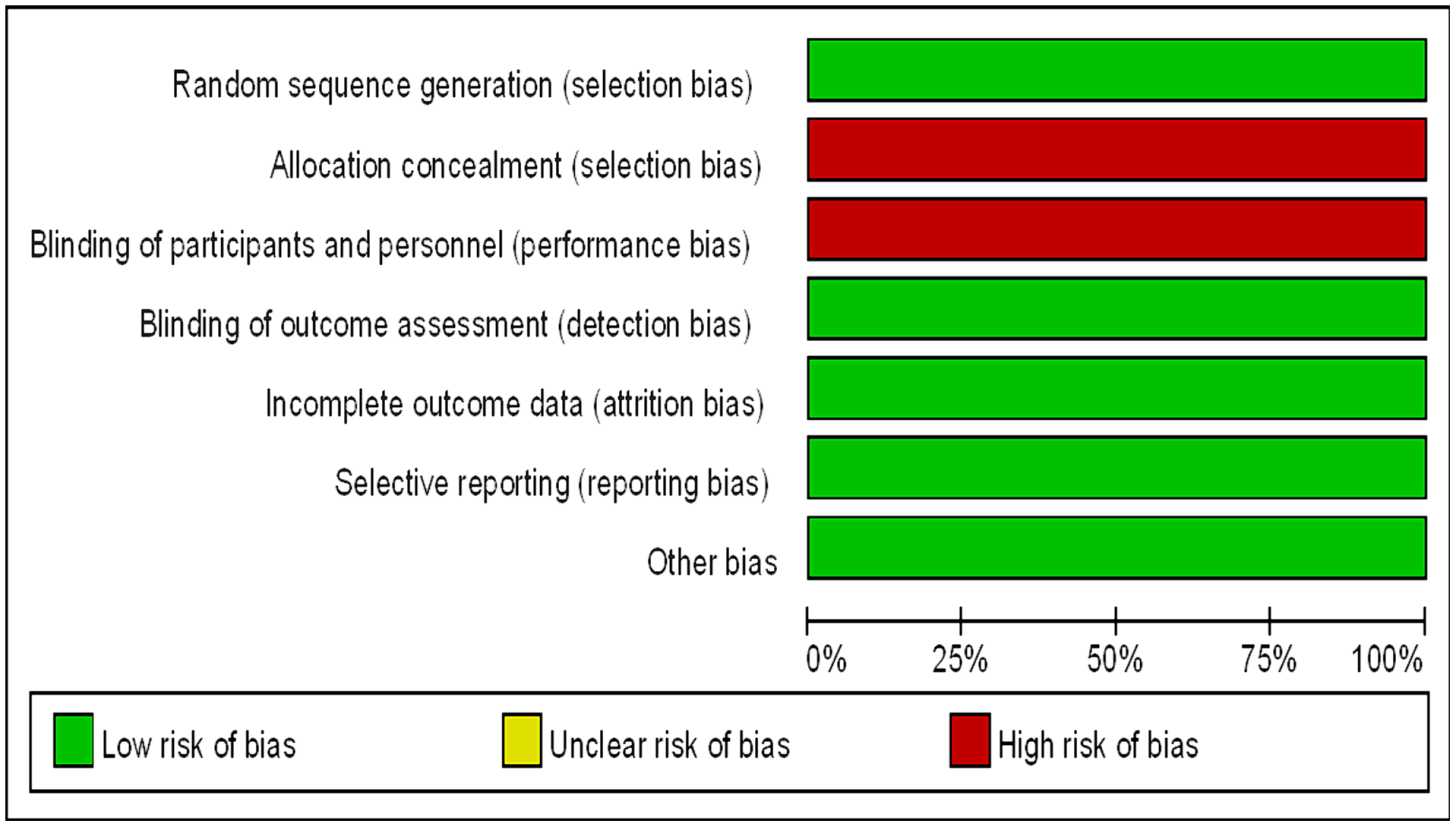
Bias risk map (the authors’ judgments of each bias risk item are expressed as a percentage across all included studies).

Assessment of the Included Studies The methodological quality of the included trials were assessed using the Cochrane Collaboration’s risk of bias tool. The risk of bias in the selected studies were independently assessed by three authors (14). The studies were rated as “low risk,” “high risk,” or “unclear risk” in specific areas, such as selection bias, performance bias, detection bias, attrition bias, and reporting bias. The endpoints reported in the published papers were compared with those listed in the study protocol or trial registry. The results are summarized in a risk of bias diagram (Figure 1).

### 2.5 Types of outcome measures

The following outcome measures were evaluated: OS, PFS, CR rates, PR rates, and AEs. For each trial, three different authors extracted data from the safety analyses. The data were obtained from each study.

### 2.6 Statistical methods

Statistical analyses were conducted using RevMan 5.3 software (provided by the Cochrane Collaboration) for analysis of the extracted data. HRs and 95% CIs were used for effect analysis. Appropriate computational models were chosen based on the type of data included (generic inversion variance for OS and PFS and dichotomous for CR, PR, and AEs). The chi-square test was employed to assess the heterogeneity among the included studies: studies with I^2^ > 50% were considered to have high heterogeneity and were analyzed using a random-effects model; studies with I^2^ ≤ 50% were considered to have low heterogeneity and were analyzed using a fixed-effects model (15).

## 3 Results

### 3.1 Selected studies

In our comprehensive literature search, 1,382 potentially relevant articles were retrieved and imported into EndNote X8 for further management. Subsequently, two researchers meticulously screened the titles and abstracts and accessed the full text of the studies whose inclusion status was uncertain to finalize the inclusion decisions. A third researcher conducted a thorough verification of the inclusion and exclusion criteria in the literature. Discrepancies encountered during the literature review process were resolved through collaborative discussion. We found and deleted 279 duplicate articles. Through a meticulous assessment of titles, abstracts, and full texts, 1,086 records were excluded because they were found to be unrelated to our study (including systematic reviews and meta-analyses, editorials, case reports, review articles, preclinical studies, retrospective studies, single-arm trials, nonrandomized trials, and ongoing studies/trials).

**Figure 2:**
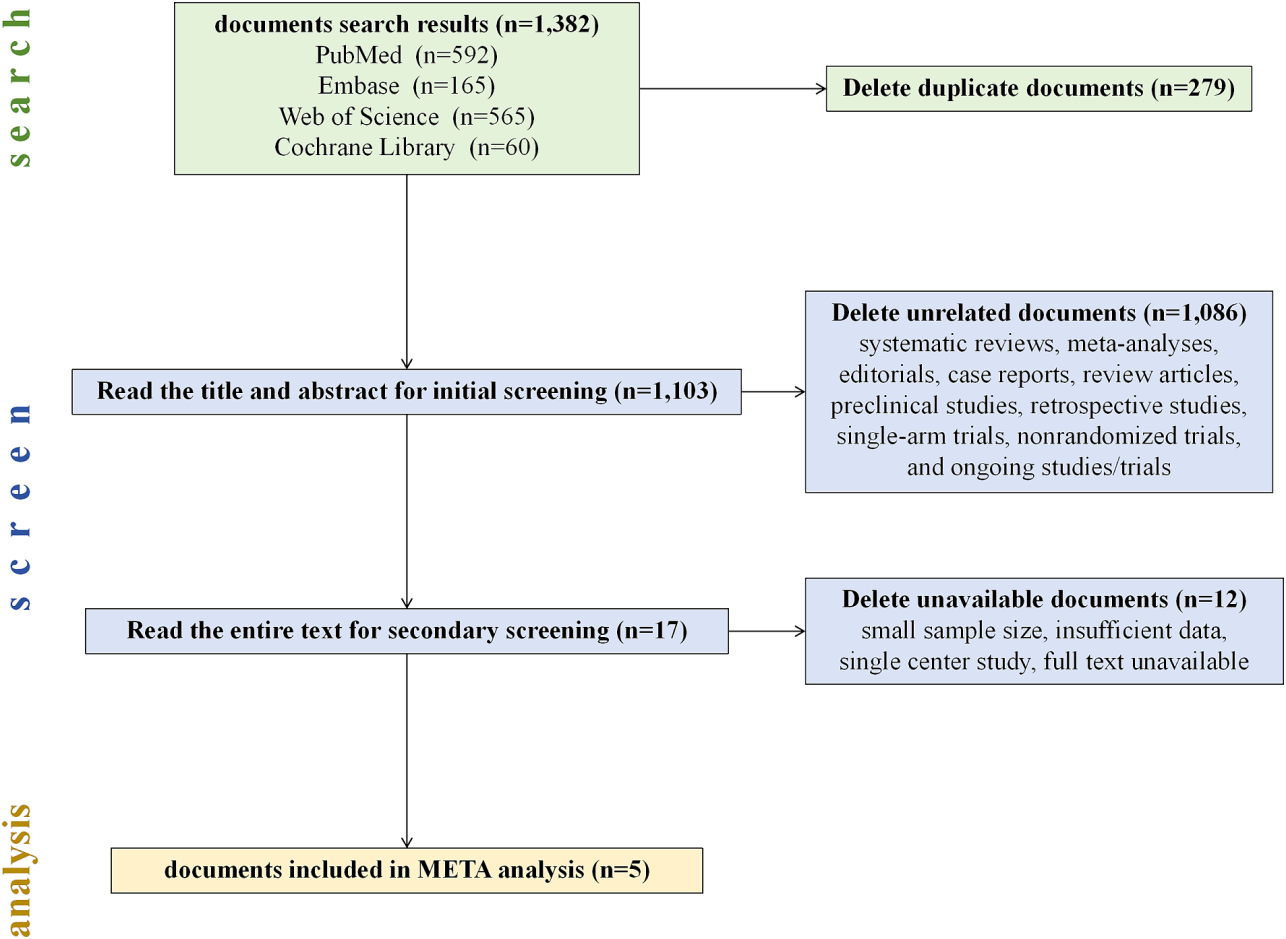
Flow chart of the literature screening process.

Ultimately, the literature review included 5 studies for meta-analysis (8–12), and the detailed search and screening flowchart is presented in Figure 2. All studies included in our analysis (Table 1) were determined to have a low risk of bias following a separate review by three authors. These studies compared immune-based combination therapy with sorafenib as a first-line treatment for patients with advanced HCC. A total of 2,989 HCC patients were included in the meta-analysis (ICIs = 1798; sorafenib = 1,191).

**Table 1:** Basic characteristics of the patients included in the study.

Documents	Year	Stage	Number of cases		Therapeutic method		Outcome measures
			Experiment	Control	Experiment	Control	
IMbrave150 (8)	2022	III	326	159	Atezolizumab + Bevacizumab	Sorafenib	PFS/OS/ORR/AEs
COSMIC-312 (9)	2022	III	250	122	Atezolizumab + Cabozantinib	Sorafenib	PFS/OS/ORR/AEs
ORIENT-32 (10)	2021	III	365	172	Sintilimab+IBI305	sorafenib	PFS/OS/ORR/AEs
HIMALAYA (11)	2022	III	393	389	Durvalumab + Tremelimumab	Sorafenib	PFS/OS/ORR/AEs
CARES-310 (12)	2023	II/III	272	271	Camrelizumab + Rivoceranib	Sorafenib	PFS/OS/ORR/AEs

### 3.2 OS and PFS

**Figure 3:**
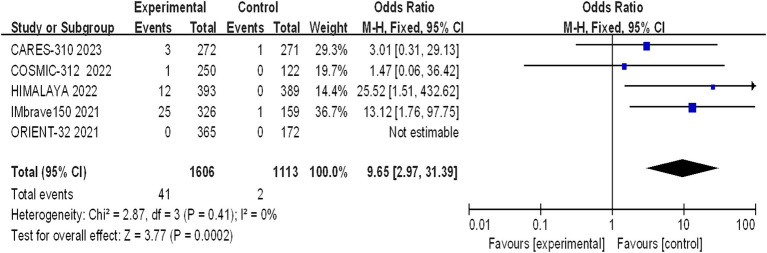
Forest plot of OS in advanced HCC patients treated with combination immunotherapy and sorafenib monotherapy.

**Figure 4:**
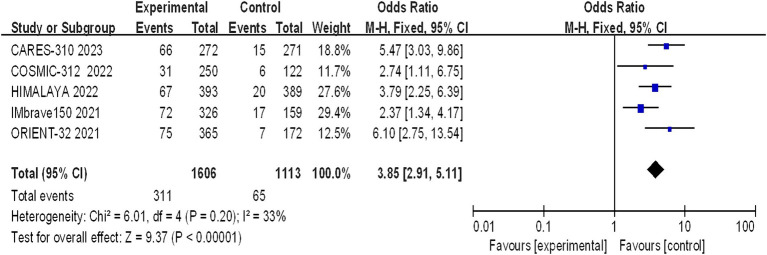
Forest plot of PFS in advanced HCC patients treated with combination immunotherapy and sorafenib monotherapy.

The OS and PFS of the included patients were reported by five studies involving a total of 2,719 patients with advanced HCC (1,606 in the immunocombination therapy group and 1,113 in the sorafenib group). Our meta-analysis demonstrated that immune combination therapy significantly prolonged patient OS (HR = 0.69, 95% CI: 0.58–0.82, *p* < 0.001) and PFS (HR = 0.64, 95% CI: 0.50–0.81, *p* < 0.001), and these differences were statistically significant (Figures 3, 4).

### 3.3 CR and PR

**Figure 5:**
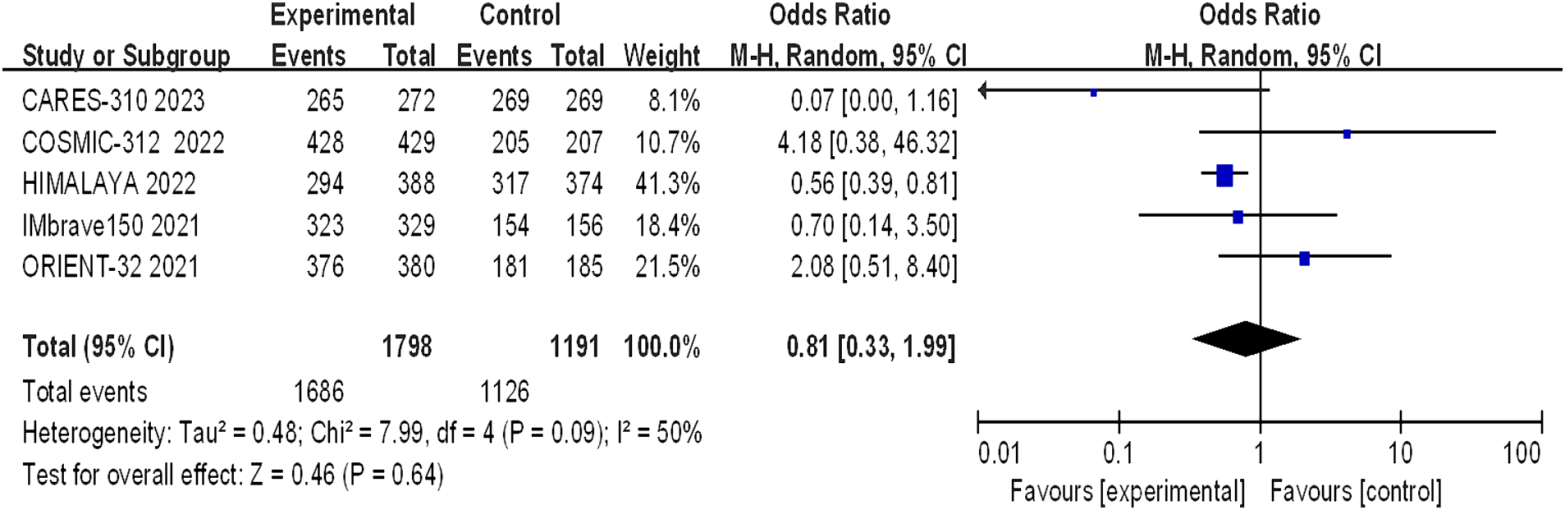
Forest plot of CR in advanced HCC patients treated with combination immunotherapy and sorafenib monotherapy.

**Figure 6:**
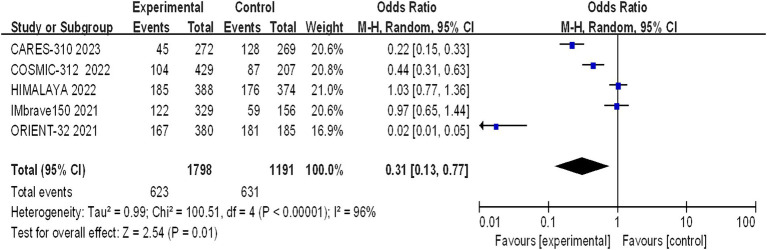
Forest plot of PR in advanced HCC patients treated with combination immunotherapy and sorafenib monotherapy.

Five studies reporting CR and PR among the included patients were included, involving a total of 2,719 patients with advanced HCC (1,606 in the immunocombination therapy group and 1,113 in the sorafenib group). Our meta-analysis revealed that immune combination therapy significantly enhanced patient CR (OR = 0.9.65, 95% CI: 2.97–31.39; *p* = 0.002) and PR (OR = 3.85, 95% CI: 2.91–5.11, *p* < 0.01) (Figures 5, 6).

### 3.4 AEs

Across the five studies, the incidence of AEs was consistent, with a cumulative total of 2,989 patients, split between 1,798 in the immune combination therapy arm and 1,191 in the sorafenib arm. The analysis was primarily anchored on treatment-related adverse events (TRAEs) categorized as grades 1–2, 3–4, and 5 by the Common Terminology Criteria for Adverse Events (CTCAE) grading schema (Table 2). The predominant adverse effects identified included hypertension, elevated transaminase levels, and proteinuria. When sorafenib was used as the reference treatment, combination therapy with camrelizumab and apatinib was associated with a greater incidence of grade ≥ 3 TRAEs (81% versus 52%). The most frequent grade 3–4 TRAEs included hypertension, hand-foot syndrome, and elevated aspartate aminotransferase and alanine aminotransferase levels. In the IMbrave150 study, the incidence of grade 3–4 TRAEs were similar between the T + A (atezolizumab plus bevacizumab) and sorafenib groups (56.5% versus 55.1%), with hypertension being the most common event (15.2% versus 12.2%). In the ORIENT-32 study, the immune combination therapy group exhibited a greater incidence of grade 3–4 TRAEs than did the sorafenib group (53% versus 45%), with hypertension (15% versus 6%), thrombocytopenia (8% versus 3%), and proteinuria (5% versus 2%) being the most frequently observed. Both groups in the COSMIC-312 trial showed a greater incidence of grade ≥ 3 TRAEs than did the sorafenib group (64% versus 46%), with the most common grade 3–4 TRAEs being elevated alanine aminotransferase (9% versus 3%), aspartate aminotransferase (9% versus 4%), hypertension (9% versus 8%), and hand-foot syndrome (8% versus 8%). However, in the HIMALAYA study, the double-immunotherapy group had a lower incidence of TRAEs (25.8% versus 36.9%) than did the sorafenib group, with hepatic events, diarrhea/colitis, and dermatitis/rash emerging as the primary immune-mediated adverse reactions.

**Table 2:** Basic characteristics of patients included in the study for reporting adverse reactions.

Documents	Year	Stage	Number of cases		Therapeutic method		Common adverse reactions in the experimental group
			Experiment	Control	Experiment	Control	
IMbrave150 (8)	2022	III	329	156	Atezolizumab + Bevacizumab	Sorafenib	Hypertension of any grade (29.8%) grade 3–4 hypertension (15.2%) fatigue of any grade (20.4%) grade V adverse reactions 4.6% (15/329)
COSMIC-312 (9)	2022	III	429	207	Atezolizumab + Cabozantinib	Sorafenib	grade 1–3 diarrhea (52%) grade 3–4 hand-foot syndrome (8%) grade V adverse reactions 12% (51/429)
ORIENT-32 (10)	2021	III	380	185	Sintilimab + IBI305	Sorafenib	Serious adverse reactions (32%) The most common grade 3 hypertension (15%) grade V adverse reactions 3% (10/380)
HIMALAYA (11)	2022	III	388	374	Durvalumab + Tremelimumab	Sorafenib	Itching of any grade (32.4%) Rash of any grade (32.4%) grade 3–4 AST increase (12.2%) grade V adverse reactions 2.3% (9/388)
CARES-310 (12)	2023	II/III	272	269	Camrelizumab + Rivoceranib	Sorafenib	grade 3 or more adverse reactions (81%) grade 3–4 hypertension (38%) grade 3–4 hand-foot syndrome (12%) grade V adverse reactions 0.37% (1/271)

#### 3.4.1 Arbitrary grade AEs

**Figure 7:**
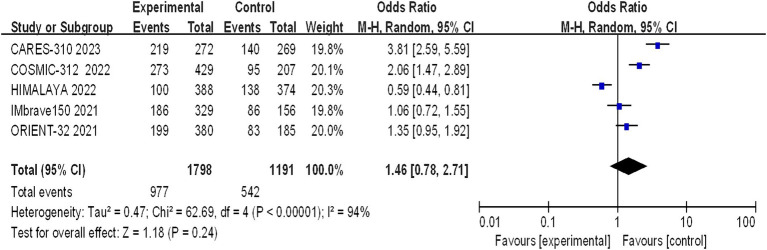
Forest plot of any grade of AEs in advanced HCC patients treated with combination immunotherapy and sorafenib monotherapy.

The incidence of arbitrary-grade AEs were documented in 5 papers, and the assessment of heterogeneity revealed significant variability across studies (I^2^ = 50%, *p* = 0.09), prompting the utilization of a random effects model for analysis. The rates of adverse events in the immunotherapy combination group and the sorafenib group were 93.72% and 94.54%, respectively. The meta-analysis revealed no statistically significant difference in the overall incidence of AEs between the experimental and control groups (OR = 0.81, 95% CI: 0.33–1.99, *p* = 0.64) (Figure 7).

#### 3.4.2 Incidence of grade 1–2 AEs

**Figure 8:**
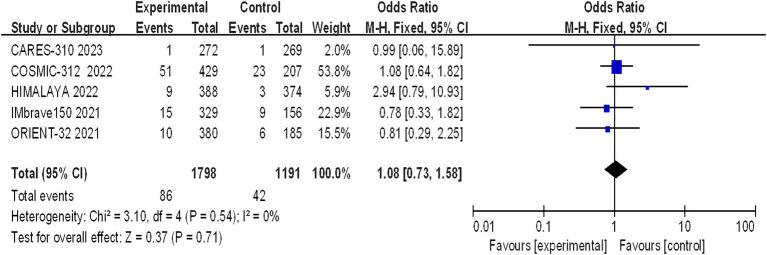
Forest plot of grade 1–2 AEs in advanced HCC patients treated with combination immunotherapy and sorafenib monotherapy.

Grade 1–2 adverse reactions were reported in all five studies, and the assessment of heterogeneity indicated significant variability among the studies (I^2^ = 96%, *p* < 0.00001), necessitating the use of a random-effects model for analysis. The meta-analysis indicated a statistically significant difference in the incidence of grade 1–2 adverse reactions between the trial group and the control group (OR = 0.31, 95% CI: 0.13–0.77; *p* = 0.01) (Figure 8).

#### 3.4.3 Incidence of grade 3–4 AEs

**Figure 9:**
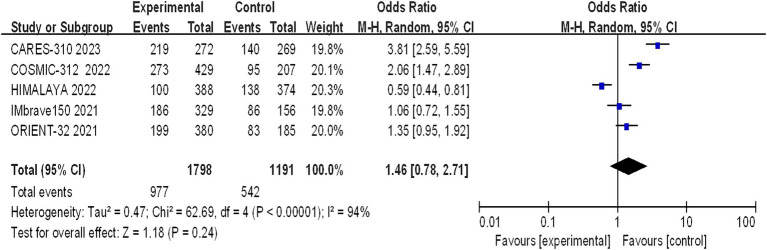
Forest plot of grade 3–4 AEs in advanced HCC patients treated with combination immunotherapy and sorafenib monotherapy.

Grade 3–4 adverse reactions were documented in all five studies, and the assessment of heterogeneity indicated significant variability among the studies (I^2^ = 94%, *p* < 0.00001), prompting the use of a random-effects model for analysis. The meta-analysis findings suggested that there were no statistically significant differences in the incidence of grade 3–4 adverse reactions between the experimental and control groups (OR = 1.46, 95% CI: 0.78–2.71; *p* = 0.24) (Figure 9).

#### 3.4.4 Incidence of grade 5 AEs

**Figure 10:**
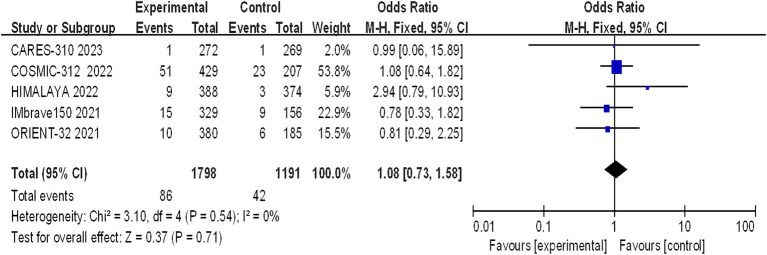
Forest plot of grade 5 AEs in advanced HCC patients treated with combination immunotherapy and sorafenib monotherapy.

Five studies reported grade 5 adverse reactions, and the heterogeneity test results indicated statistical heterogeneity between studies (I^2^ = 0, *p* = 0.54). This difference was analyzed using a fixed effects model. This indicates that there were no statistically significant differences in the incidence of grade 5 adverse reactions between the experimental group and the control group (I^2^ = 1.08, 95% CI: 0.73–1.58, *p* = 0.71) (Figure 10).

## 4 Discussion

Since 2007, targeted therapies such as sorafenib, lenvatinib, regorafenib, and cabozantinib have emerged as pivotal agents in oncology. Despite their introduction, clinical practice has highlighted that the response rates to these drugs are often insufficient, and they are frequently associated with a concerning array of adverse effects. This has underscored the necessity for the exploration of more effective and better-tolerated treatment modalities. In recent years, substantial progress has been made in the systemic management of advanced hepatocellular carcinoma (HCC) (11). Immunological monotherapy has demonstrated limited efficacy, with a minority of HCC patients responding favorably. The expansion of therapeutic options and the development of combination regimens for initial treatment of advanced HCC have presented significant challenges for healthcare professionals. To evaluate the efficacy and safety of ICI-based immunocombination therapy versus sorafenib monotherapy in advanced HCC patients, this study conducted a comprehensive systematic analysis. In our investigation, we meticulously selected five high-caliber, foreign phase III RCTs by examining English databases to appraise the efficacy and safety of immune-based combination therapy versus sorafenib for advanced hepatocellular carcinoma, utilizing OS, PFS, CR, PR, and AEs as evaluating indicators.

### 4.1 Efficacy

Our meta-analysis aimed to delineate the clinical efficacy of immune-based combination therapy versus sorafenib in the treatment of advanced HCC. The analysis included five pivotal studies: CARES-310, COSMIC-312, HIMALAYA, IMbrave150, and ORIENT-32 collectively enrolling 2,719 patients with HCC. The meta-analysis revealed the encouraging efficacy: patients treated with immune-based combination therapy demonstrated significantly prolonged median OS and PFS, along with elevated rates of CR and PR. Notably, the CR rate in HCC patients treated with immune-based combination therapy was more than 14 times greater than that in patients receiving sorafenib monotherapy, indicating the potential for long-term benefits and curative outcomes in certain advanced HCC patients. A successful phase III trial for the combination of atezolizumab and bevacizumab (the IMbrave150 trial) in advanced hepatocellular carcinoma is groundbreaking because nivolumab and pembrolizumab, both programmed cell death-1 (PD-1) antibodies, have failed to show efficacy as first-and second-line therapeutics, respectively, in phase III clinical trials. Kudo M described the scientific rationale for the combination of PD-1/PD-L1 antibodies plus VEGF inhibitors (16). The success of the phase III IMbrave150 study suggest that the tumor microenvironment was changed by bevacizumab, enabling greater responses to the immune checkpoint blockade, as hypothesized. In 2024, based on the IMbrave-150 and HIMALAYA trial, NCCN recommended the combination of atezolizumab plus bevacizumab (for C-P Class A HCC) and the combination of tremelimumab-actl plus durvalumab are category 1 preferred first-line systemic therapy options for patients with advanced HCC. At disease progression, Sorafenib and Lenvatinib could be used as second-line strategy, followed by Cabozantinib or Regorafenib in further treatment lines, if feasible (17).

### 4.2 Safety

The safety profile of the interventions was evaluated through an assessment of all-grade adverse events and treatment-related adverse events. In this meta-analysis, the incidence of AEs was substantial in both the immune-based combination therapy group and the sorafenib group, with more than 90% of patients experiencing any grade of AEs.

A statistically significant discrepancy was noted in the incidence of grade 1–2 adverse reactions between the treatment and control groups, with the majority of symptoms being mild. However, the differences in the incidence of grade 3–4 (*p* = 0.24) and grade 5 (*p* = 0.71) adverse reactions were not statistically significant. The incidence of life-threatening grade 5 adverse reactions is a clinical concern, with the rates observed in the CARES-310, COSMIC-312, HIMALAYA, IMbrave150, and ORIENT-32 studies being 0.37% (1/271) vs. 0.37% (1/269), 12% (51/429) vs. 11% (23/207), 2.3% (9/388) vs. 0.8% (3/374), 4.6% (15/329) vs. 5.8% (9/156), and 3% (10/380) vs. 3% (6/185), respectively. The meta-analysis revealed no statistically significant difference between the two groups (OR 1.08, 95% CI: 0.73–1.58, *p* = 0.71), suggesting that combination therapy does not increase mortality but should be cautiously administered in clinical practice. The frequency of hypertension and hypothyroidism was markedly greater in the immunocombination therapy cohort than in the sorafenib cohort. The adverse effects most commonly observed with sorafenib include hypertension, hand-foot syndrome, diarrhea, fatigue, and proteinuria. In the context of monotherapy with immunotherapy, the phase III HIMALAYA study reported a lower incidence of grade ≥ 3 treatment-related adverse events (TRAEs) than did the sorafenib study (25.8% vs. 36.9%), yet the incidence of serious adverse events was greater in the immunotherapy group than in the sorafenib control group (17.5% vs. 9.4%). The effects of TRAEs on survival in patients with advanced malignancies remain elusive (18). Immuno-based combination regimens are engineered to optimize efficacy, yet they may cause a commensurate increase in toxicity, complicating the diagnostic and therapeutic management of adverse effects. Clinicians should be cognizant that the concomitant use of CTLA-4 inhibitors with PD-1/PD-L1 inhibitors may precipitate immune-related adverse events (irAEs) at an accelerated timeline and with intensified severity, particularly in the sphere of palliative care, where the preservation of patient welfare assumes primacy (18).

### 4.3 Limitations

This meta-analysis has both notable strengths and limitations. Its strengths derive from the rigorous inclusion of phase III randomized controlled trials (8–12) and the large sample size (2,719 participants, comprising 1,606 in the immune-based combination group and 1,113 in the sorafenib monotherapy group), which allows for a comprehensive assessment of both clinical efficacy and safety outcomes. However, interpretation of the meta-analysis results should be approached with caution. it is important to acknowledge the limitations of our study. First, we were unable to obtain treatment information for individual patients, and the comprehensive data included in the analysis were extracted from clinical trial results. Second, the diversity of immune-based combination therapies included in the trials, such as atezolizumab plus bevacizumab, atezolizumab plus cabozantinib, sintilimab plus IBI305, durvalumab plus tremelimab, and camrelizumab with apatinib, requires acknowledgment that their individual efficacy and safety profiles are distinct and not directly comparable, a factor that must be taken into account when interpreting the collective findings. Third, demographic variations across the studies should be considered. For example, a lower proportion of Asian patients were diagnosed with COSMIC-312 than with CARES-310, HIMALAYA, IMbrave150, or ORIENT-32, as detailed in Table 1 (8–12). Conversely, IMbrave150 and ORIENT-32 had a greater proportion of HBV-positive patients than COSMIC-312 and HIMALAYA. Clinical outcome heterogeneity is another critical point, with COSMIC-312 reporting a final PFS analysis but an interim OS analysis and being the sole trial to not show a significant OS benefit. Notably, high CR rates were observed for immune-based combination therapies, but they were not uniformly distributed, primarily in the HIMALAYA and IMbrave150 studies. As illustrated in Figure 5, only one CR case was observed in the COSMIC-312 cohort, while none were observed in the ORIENT-32 cohort, and no improvement in PFS was noted in the HIMALAYA cohort. Fourth, The search was limited to English-language literature, potentially resulting in the exclusion of relevant studies and limiting the generalizability of the results. Fifth, the characteristics of the study population, such as Child–Pugh class and Eastern Cooperative Oncology Group performance status, may influence the efficacy and safety of PD-1/PD-L1 inhibitors for advanced HCC, but can not be sufficiently extracted, which fails to perform further subgroup analysis. Finally, while the current study corroborated that immune combination therapy extends OS and PFS and enhances CR and PR rates, heterogeneity existed within the dataset. Due to the limited number of included articles, subgroup analyses to validate the consistency of these findings across various dosing regimens, types of viral infections, and other factors were not conducted. The research in this study was rigorously screened for the inclusion of literature to minimize the occurrence of bias, but The strict inclusion criteria of our systematic review allowed for the selection of only II and III studies. Despite these limitations, the study also ended up including a sample size, and we believe that this study will contribute to a better understanding of the current reality of advanced HCC treatment.

### 4.4 Future perspectives

Atezolizumab in combination with bevacizumab represents the first therapy demonstrated to be superior to sorafenib monotherapy, and this regimen is now established as the standard of care for the initial treatment of advanced HCC in the majority of patients, particularly those without prohibitive contraindications and regardless of financial considerations, heralding a new era in the management of HCC (8). Furthermore, other immune-based combination strategies have been shown to prolong OS and PFS and increase the rates of CR and PR, as confirmed by a meta-analysis conducted by Rizzo et al. (19). A multitude of contemporary studies have also substantiated that immune-based combinations are a viable approach for the management of advanced HCC (20, 21). Currently, in the absence of direct comparisons between first-line immune-based therapies, clinicians must judiciously select treatments based on nuanced inclusion criteria and demographics across different clinical trials, factoring not only drug efficacy but also a spectrum of variables, including adverse drug reactions, patient comorbidities, and pharmacoeconomics. In this rapidly evolving landscape, patient selection remains key, and predictive biomarkers are urgently required to allocate patients to the best treatment, avoiding toxicities to those who are not expected to derive therapeutic benefit (22). Given that tumoural PDL1 expression only correlates with the objective response to nivolumab in patients with aHCC, optimal predictive biomarkers of response still need to be identified (23).

### 4.5 Questions

Many questions remain unresolved. The presence of underlying liver disease and poor chemosensitivity pose major treatment challenges in the management of HCC. First, the paucity of studies examining second-line therapy following ICIs necessitates careful consideration of the optimal sequence of treatment, which is a subject of debate. Moreover, safety is paramount in the context of palliative care, particularly from the patient’s perspective. Although all trials have documented manageable toxicity in HCC patients treated with immune-based combination therapies, several considerations merit emphasis. Given that HCC patients frequently present with coexisting conditions (e.g., cirrhosis, metabolic abnormalities) and compromised liver function, it is imperative to closely monitor patient comorbidities and to consider potential contraindications to ICI use in this patient population. Additionally, the absence of validated biomarkers to predict therapeutic efficacy is a significant challenge, as only a subset of HCC patients benefit from immunotherapy (24). Consequently, a deeper understanding of potential biomarkers, such as PD-L1 expression, tumor mutational burden (TMB), microsatellite instability (MSI) status, and the gut microbiota, is of critical importance. For instance, HCC etiology is believed to influence the response to ICIs, and preclinical studies have demonstrated intrinsic resistance to anti-PD-1 therapy in a mouse model of nonalcoholic steatohepatitis-associated hepatocellular carcinoma (NASH-HCC), corroborated by a seminal study indicating reduced efficacy of ICIs in HCC unrelated to viral etiology (25, 26). Unfortunately, there has not been a single predictive biomarker (except for elevated serum AFP for ramucirumab) linked to the therapeutic response to any therapeutic agent (27). Hence, there is an urgent need to improve the effectiveness of immunotherapy in HCC through biomarker-directed therapy, patient stratification and careful combination selection. Various driver mutations have been implicated in the pathogenesis of HCC, including TP53 mutations associated with HBV infection and CTNNB1 mutations related to alcoholism (28–30). Furthermore, insights into the tumor microenvironment (TME) may elucidate additional strategies for HCC immunotherapy. Therapeutic regimens, including immune-based combinations, could reshape the TME of HCC by eliciting adaptive responses in cellular components that reflect the altered transcriptome and proteome. Under therapeutic pressure, these adaptive responses could not only improve survival, progression, and metastasis rates in HCC but also offer novel therapeutic options, including immunotherapies and antiangiogenic agents. There is an urgent need to advance precision HCC immunotherapy, as the etiology of HCC may play a significant role. Future clinical trials should stratify patients by taking into account the tumor biology and the patient clinical characteristics, and the coming years will determine how these emerging drugs and immune-based combinations will influence the treatment landscape of liver cancer. Overall, the integration of immunotherapy, antiangiogenesis, and second-line treatment options represents a comprehensive approach to managing advanced HCC, with the potential to improve patient outcomes and redefine the treatment landscape for this challenging disease (31).

## 5 Conclusion

The current meta-analysis underscores that immune-based combination therapies represent a formidable therapeutic strategy for individuals with unresectable or moderately advanced HCC, exhibiting marked superiority over sorafenib monotherapy in the initial treatment setting. The significantly elevated CR rate observed with immune-based combinations, which was more than 14 times greater than that achieved with sorafenib monotherapy, implies the potential for curative outcomes in select patients with advanced liver cancer. In formulating an optimal first-line treatment regimen for HCC, a meticulous weighing of therapeutic efficacy against the risk of toxicity is essential. ICI-based therapeutic strategies, particularly the combination of ICIs and targeted agents, are promising for the treatment of advanced HCC. However, the optimal treatment strategy and timing of ICI administration in HCC remain challenging.

Forecasting the response to immunotherapy and pinpointing the patient subgroups most susceptible to benefit from these interventions will constitute the focal point of future studies.

## Data availability statement

The original contributions presented in the study are included in the article/supplementary material, further inquiries can be directed to the corresponding author.

## Author contributions

MS: Writing – original draft, Writing – review & editing. YW: Writing – review & editing. MC: Writing – review & editing. SF: Writing – review & editing. GL: Writing – review & editing. HR: Writing – review & editing.
